# Integrated multi-omics analysis reveals gut microbiota-associated placental metabolic and transcriptomic alterations in placenta previa

**DOI:** 10.1128/msystems.00687-26

**Published:** 2026-07-15

**Authors:** Yiwen Zhang, Aiqing Zhang, Qiuyang Li, Yuhua Luo, Qian Gao, Xuejiao Li, Xiujie Li, Yi Zhang, Chengfang Xu, Zhe Li

**Affiliations:** 1 Department of Obstetrics, The Third Affiliated Hospital of Sun Yat-Sen University 144991, Guangzhou, Guangdong, China; 2 Department of Hepatobiliary Surgery, The Third Affiliated Hospital of Sun Yat-Sen University 144991, Guangzhou, Guangdong, China; Georgia Institute of Technology, Atlanta, Georgia, USA

**Keywords:** placenta previa, gut microbiota, oral microbiota, vaginal microbiota, short-chain fatty acids, cholinergic synapse pathway

## Abstract

Placenta previa is a major obstetric complication, yet its potential association with maternal microbiota-associated biological alterations remains largely unexplored. This study aimed to investigate microbiota-associated metabolic and transcriptomic alterations in placenta previa using an integrated multi-omics approach. A total of 72 third-trimester pregnant women (28 with complete placenta previa and 44 controls) were enrolled. Maternal gut, oral, and vaginal microbiota were profiled using 16S rRNA gene sequencing, while placental tissues underwent targeted short-chain fatty acid (SCFA) metabolomic profiling and RNA sequencing. Multi-omics integration, ecological network analysis, and pathway enrichment analyses were performed to identify microbiota-associated biological alterations linked to placenta previa. Global microbial diversity remained largely preserved across maternal body sites in placenta previa. However, gut microbiota demonstrated prominent ecological network remodeling and was associated with coordinated placental SCFA and transcriptomic alterations. Several placental SCFAs, including acetic acid, propionic acid, isobutyric acid, and isovaleric acid, exhibited increased abundance trends in placenta previa. Integrated metabolomic and transcriptomic analyses convergently identified cholinergic synapse signaling as a shared altered pathway. In addition, cholinergic signaling-related genes, including CHRNB4 and GNB5, demonstrated coordinated associations with altered placental SCFAs and specific gut microbial taxa. These findings suggest the existence of a potential gut microbiota–placental metabolite–transcriptome axis associated with placenta previa. Multi-omics convergence on cholinergic synapse signaling highlights a potential biological pathway linking gut microbiota-associated metabolic alterations with placental transcriptomic remodeling in placenta previa.

**IMPORTANCE:**

Placenta previa is a major obstetric complication with poorly understood biological mechanisms beyond established anatomical and clinical risk factors. This study provides the first integrated multi-omics investigation linking maternal microbiota, placental short-chain fatty acids (SCFAs), and placental transcriptomic alterations in placenta previa. Rather than identifying broad microbial ecosystem disruption, we observed coordinated ecological remodeling of gut microbiota together with altered placental SCFA profiles and transcriptomic pathways. Notably, metabolomic and transcriptomic analyses convergently implicated cholinergic synapse signaling as a shared altered pathway associated with placenta previa. These findings support the existence of a potential gut microbiota–placental metabolite–transcriptome axis and provide new biological insights into microbiota-associated signaling alterations in placenta previa.

## INTRODUCTION

The maternal microbiota has attracted increasing research attention in recent years, as it undergoes dynamic shifts throughout pregnancy and has been implicated in various pregnancy-related disorders. Numerous studies have suggested associations between alterations in maternal microbiota and adverse obstetric outcomes (1–3). Importantly, these microbial changes are not static but rather dynamic processes that may influence maternal and fetal health (4).

Placenta previa is the complete or partial covering of the internal os of the cervix with the placenta. It is a major risk factor for postpartum hemorrhage and can lead to morbidity and mortality of the mother and neonate (5). Existing research has primarily focused on the maternal and fetal clinical outcomes (6, 7) and risk factors (8, 9) associated with placenta previa, while the potential role of the microbiota in its pathogenesis has received no attention.

Previous evidence has suggested potential associations between maternal microbial communities, particularly gut (10, 11) and oral (12) microbiota, and the placental immune regulation (13, 14). In parallel, increasing evidence indicates that microbiota-derived metabolites, especially short-chain fatty acids (SCFAs), may participate in host metabolic regulation, immune signaling, and maternal-fetal communication during pregnancy (15–17). However, whether coordinated microbiota-associated metabolic and placental transcriptomic alterations are involved in placenta previa remains unclear.

To address this knowledge gap, we performed an integrated multi-omics investigation involving maternal gut, oral, and vaginal microbiota together with placental SCFA metabolomics and placental transcriptomic profiling in pregnancies complicated by placenta previa. By combining microbial ecological analysis with placental metabolomic and transcriptomic pathway analyses, we aimed to identify potential microbiota-associated biological signatures and shared signaling pathways associated with placenta previa. This study provides a novel systems-level perspective on microbiota–placenta interactions in placenta previa and identifies candidate pathways for future mechanistic investigation.

## MATERIALS AND METHODS

### Study design, participants, and sample collection

Participants were recruited between 1 May 2025 and 30 September 2025. A total of 72 women with singleton pregnancies in the third trimester who underwent elective cesarean section were enrolled in this study, comprising 28 women diagnosed with complete placenta previa and 44 pregnant women with normal placentation who were scheduled for cesarean section due to a scarred uterus or non-medical indications. Written informed consent was obtained from all participants prior to enrollment, and the study protocol was approved by the institutional ethics committee.

The exclusion criteria were as follows: (i) gastrointestinal diseases or a family history of gastrointestinal disorders; (ii) antibiotic use within 30 days prior to sample collection; (iii) hypertension, diabetes mellitus, hyperthyroidism, hypothyroidism, autoimmune diseases, or other endocrine and metabolic disorders; (iv) a history of blood transfusion, organ transplantation, or immunotherapy; (v) active vaginal or sexually transmitted infections, including bacterial vaginosis, vulvovaginal candidiasis, trichomoniasis, Chlamydia trachomatis, Ureaplasma urealyticum, or Neisseria gonorrhoeae infection, as well as symptoms such as vaginal itching or abnormal discharge; (vi) oral diseases or recent dental treatment/professional oral cleaning within 30 days prior to sample collection; and (vii) placenta accreta spectrum. None of the participants reported regular probiotic supplementation during pregnancy.

**Fig 1:**
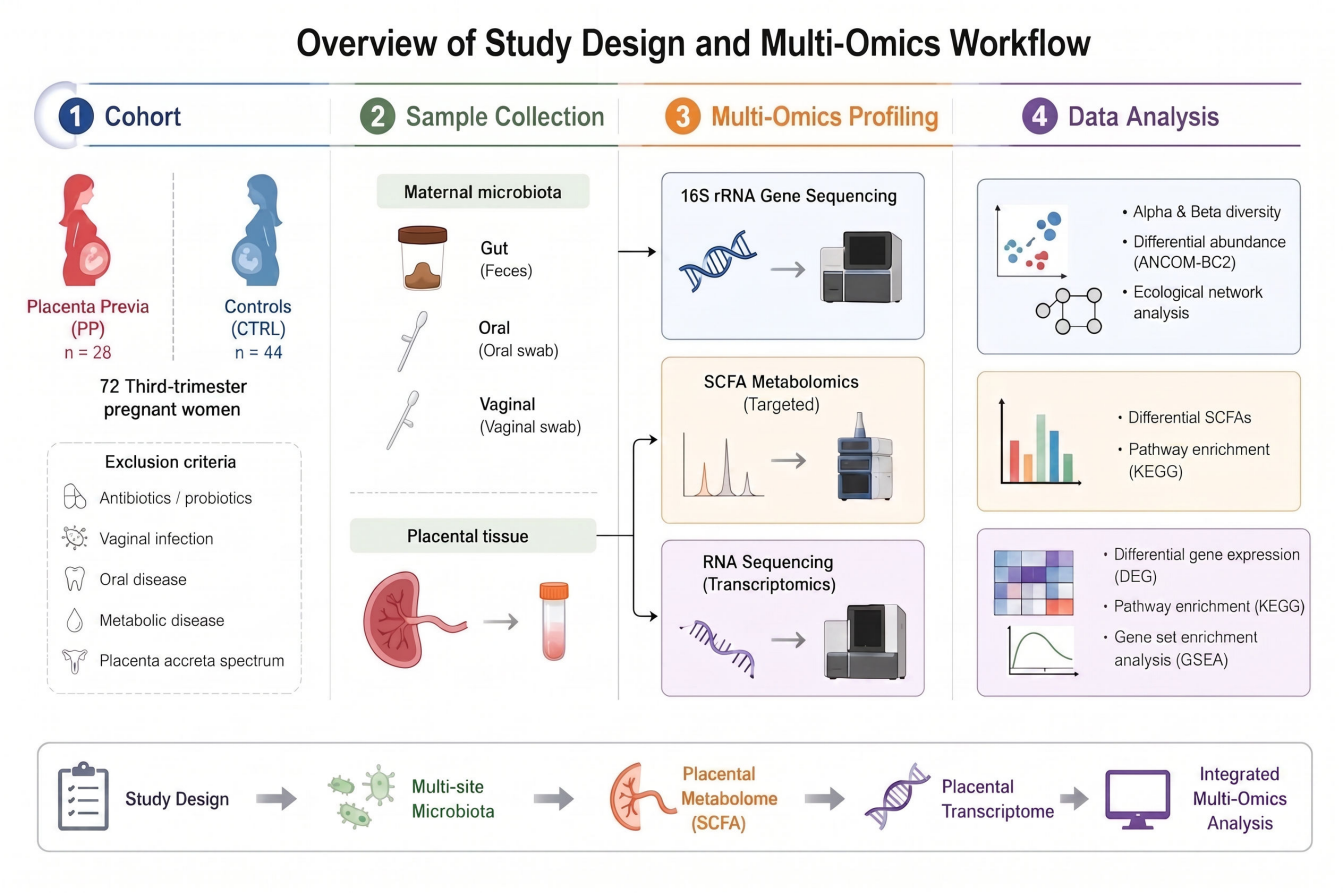
Flow chart of our study.

An overview of the study workflow is shown in Fig. 1. For clarity throughout the manuscript, microbiome samples from the gut, oral cavity, and vagina were abbreviated as G, O, and V, respectively. Placental tissue samples used for metabolomic and transcriptomic profiling were abbreviated as P. Samples from the placenta previa group were labeled as “PP,” whereas samples from the control group were labeled as “CTRL.” Baseline clinical characteristics of the participants are summarized in Table 1.

**TABLE 1:** Definition of study group symbols

Symbol	Meaning
G	Gut
O	Oral cavity
V	Vagina
P	Placenta previa
CTRL	Control group
PP	Placenta previa group

### Fecal collection

Fresh fecal samples were collected from participants prior to cesarean section using sterile fecal collection containers. Samples were obtained in the hospital and immediately transferred on ice to the laboratory, where they were stored at −80°C within 30 min of collection (18, 19).

### Vaginal secretion collection

Vaginal secretions were collected from participants prior to cesarean section. Participants were instructed to abstain from sexual activity, avoid cleansing of the vulva or vagina, and refrain from using vaginal medications within 48 h prior to sample collection. Before sampling, all participants underwent laboratory testing of vaginal discharge to exclude bacterial vaginosis (Nugent score ≥ 7), vulvovaginal candidiasis, trichomoniasis, Chlamydia trachomatis, Ureaplasma urealyticum, and Neisseria gonorrhoeae infections. A sterile vaginal speculum was used to expose the vagina, and sterile cotton swabs were used to collect vaginal secretions from the mid-lateral vaginal wall. The collected specimens were immediately stored at −80°C until subsequent 16S rRNA gene sequencing analysis.

### Oral sample collection

Oral samples were collected from participants prior to cesarean section. Participants were instructed to refrain from tooth brushing, eating, or drinking for at least 30 min before sampling. Sterile swabs were used to thoroughly sample the teeth, gums, saliva, and bilateral buccal mucosa. Oral samples were obtained in the hospital and transferred on ice to the laboratory, where they were stored at −80°C within 30 min of collection.

### Placental sample collection

Placental tissue samples were collected under sterile conditions immediately after delivery. After careful removal of the amniotic membrane, the placental tissue approximately 3 cm from the umbilical cord insertion site was exposed. Surface blood was gently rinsed off repeatedly using sterile saline. A single placental tissue sample, approximately 1 cm³ in volume, was aseptically excised from the maternal side of the placenta. The sample was immediately transferred on ice to the laboratory and stored at −80°C within 30 min of collection.

### Experimental procedure

#### 16S rRNA gene sequencing and microbiome data processing

Microbial genomic DNA was extracted from fecal samples using a magnetic bead-based Fecal Genomic DNA Extraction Kit (TianGen, Beijing, China) according to the manufacturer’s instructions. DNA from vaginal and oral swabs was extracted using the cetyltrimethylammonium bromide (20). Placental tissue was not subjected to 16S rRNA gene sequencing and was used only for metabolomic and transcriptomic profiling.

The V3–V4 region of the bacterial 16S rRNA gene was amplified using primers 341F (CCTAYGGGRBGCASCAG) and 806R (GGACTACNNGGGTATCTAAT). Sequencing libraries were constructed and sequenced on an Illumina MiSeq platform using paired-end sequencing.

Raw sequencing data were processed using QIIME 2 (version 2026.1) (21). Paired-end reads were imported into QIIME 2, and sequence quality profiles were inspected using the demux summarize function. Paired-end reads were joined prior to Deblur processing, and joined sequences were denoised using Deblur (22) with a trim length of 410 bp selected based on sequence quality profiles to generate amplicon sequence variants (ASVs). Taxonomic annotation of representative ASVs was performed using a pre-trained SILVA 138.2 Naïve Bayes classifier in QIIME 2 (23). Microbiome analyses were conducted at the ASV level or summarized at the genus level.

### Targeted SCFA metabolomics by GC-MS/MS

Targeted SCFA metabolomic profiling was performed by MetWare (http://www.metware.cn/) using the Agilent 7890B-7000D GC-MS/MS platform. Briefly, SCFAs were extracted from 50 mg of thawed placental tissue using 0.5% phosphoric acid and methyl tert-butyl ether containing internal standards. After vortexing, sonication, and centrifugation, the supernatant was subjected to GC-MS/MS analysis using an Agilent 7890B-7000D system equipped with a DB-FFAP column. The injection volume was 1 μL (split ratio 5:1), and the oven temperature was programmed from 50°C to 220°C. Helium was used as the carrier gas at a flow rate of 1.2 mL/min, and all samples were analyzed in multiple reaction monitoring mode. Metabolite identification and quantification were performed using authentic standards and the MetWare in-house database.

### RNA-seq library preparation and sequencing

Total RNA was extracted from placental tissue samples and quantified using a Qubit fluorometer. RNA integrity was assessed using a Qsep400 biofragment analyzer. mRNA libraries were constructed by poly(A) enrichment using oligo(dT) magnetic beads, followed by fragmentation, reverse transcription, adapter ligation, and PCR amplification according to standard protocols.

Sequencing was performed on the MGI DNBSEQ-T7 platform by MetWare Biotechnology Co., Ltd. (Wuhan, China). Raw sequencing reads were filtered using fastp to remove low-quality reads and adapter sequences. Clean reads were aligned to the reference genome using HISAT2, and gene expression quantification was performed using featureCounts.

Differential gene expression analysis between groups was conducted using DESeq2 (24) with Benjamini–Hochberg correction. Genes with adjusted *P* values <0.05 were considered differentially expressed. Functional enrichment analyses, including KEGG pathway enrichment, were performed using clusterProfiler.

### Alpha diversity

Alpha diversity was calculated in QIIME 2 using the observed diversity, Chao1, Shannon, and Simpson indices to evaluate microbial richness and diversity. Prior to alpha and beta diversity analyses, feature tables were rarefied to an even sequencing depth of 17,000 reads per sample. Differences in alpha diversity between groups were assessed using the Wilcoxon rank-sum test.

### Beta diversity

Beta diversity was evaluated using weighted UniFrac distances in QIIME 2 and visualized by principal coordinates analysis (PCoA). Permutational multivariate analysis of variance (PERMANOVA) was performed to assess differences in community composition between groups, and permutational analysis of multivariate dispersions (PERMDISP) (25) was used to evaluate differences in group dispersion.

### Differential abundance analysis

Differential abundance analysis was performed using ANCOM-BC2 (26) with adjustment for potential clinical confounders where appropriate. Taxa with adjusted *q* values <0.05 were considered differentially abundant. Sensitivity analyses implemented in ANCOM-BC2 were additionally evaluated to assess the robustness of differential abundance findings. Additional prevalence-based sensitivity analyses were performed using stricter filtering thresholds, including requiring taxa to be present in at least 20% of samples and to have a minimum total count threshold of 20 across samples, to evaluate the robustness of differential abundance findings.

### Association analysis

Spearman correlation analysis with Benjamini–Hochberg false discovery rate (FDR) correction was performed to evaluate associations among microbial taxa, placental metabolites, and transcriptomic features. Correlation analyses and data visualization were conducted in R.

### Statistical analysis

Continuous variables are presented as mean ± standard deviation or median (interquartile range), as appropriate. Categorical variables are presented as counts and percentages. Differences between groups were evaluated using Student’s *t* test or Wilcoxon rank-sum test for continuous variables and chi-square test or Fisher’s exact test for categorical variables, as appropriate.

Multiple testing correction was performed using the Benjamini–Hochberg FDR method, and adjusted *q* values <0.05 were considered statistically significant where applicable. Statistical analyses were performed using R.

## RESULTS

Baseline maternal and fetal characteristics are summarized in Table 2. No significant differences were observed between the placenta previa (PP) and control (CTRL) groups with respect to maternal age, height, weight, BMI, gravidity, parity, mode of conception, or infant sex distribution (all *P* > 0.05). Notably, maternal BMI was comparable between the two groups (25.0 ± 2.7 vs 26.1 ± 2.8 kg/m², *P* = 0.133).

**TABLE 2:** Baseline maternal and fetal characteristics of each group^*a*^

Characteristic	PP (*n* = 28)	CTRL (*n* = 44)	*P* value
Mode of conception, *n* (%)			0.553
NC	22 (78.6%)	37 (84.1%)	
IVF	6 (21.4%)	7 (15.9%)	
Infant sex, *n* (%)			0.936
Male	15 (53.6%)	24 (54.5%)	
Female	13 (46.4%)	20 (45.5%)	
Gestational age at delivery, days	260 (249, 267)	272 (268, 277)	<0.001
Maternal age, years (±SD)	33.8 (3.0)	32.0 (4.3)	0.07
Mean height, cm (±SD)	157.7 (5.1)	159.0 (4.6)	0.301
Mean weight, kg (±SD)	62.2 (7.3)	65.9 (7.2)	0.052
BMI, kg/m^2^ (±SD)	25.0 (2.7)	26.1 (2.8)	0.133
Gravidity	2 (1, 4)	2 (2, 3)	0.311
Parity	2 (1, 2)	2 (1, 2)	0.287
Neonatal birth weight, g (±SD)	2,743.7 (540.7)	3,213.3 (349.7)	<0.001
Birth length	48 (46, 50)	49 (48, 50)	0.058
1-min Apgar score	10 (10, 10)	10 (10, 10)	0.100
5-min Apgar score	10 (10, 10)	10 (10, 10)	0.248

^*a*^ Data are presented as mean ± standard deviation (SD) (normally distributed continuous variables), median (interquartile range [IQR]) (non-normally distributed continuous variables), or number (percentage) (categorical variables). NC, natural conception; IVF, *in vitro* fertilization. *P* values: derived from independent sample *t*-test (normally distributed continuous variables), Mann-Whitney *U* test (non-normally distributed continuous variables), or chi-square test (categorical variables).

Compared with the CTRL group, the PP group had a significantly lower gestational age at delivery (260 [249–267] vs 272 [268–277] days, *P* < 0.001) and lower neonatal birth weight (2,743.7 ± 540.7 vs 3,213.3 ± 349.7 g, *P* < 0.001). No significant differences were observed in birth length or Apgar scores between the two groups.

### Characterizing the composition of gut, oral, and vaginal microbiota in two groups

**Fig 2:**
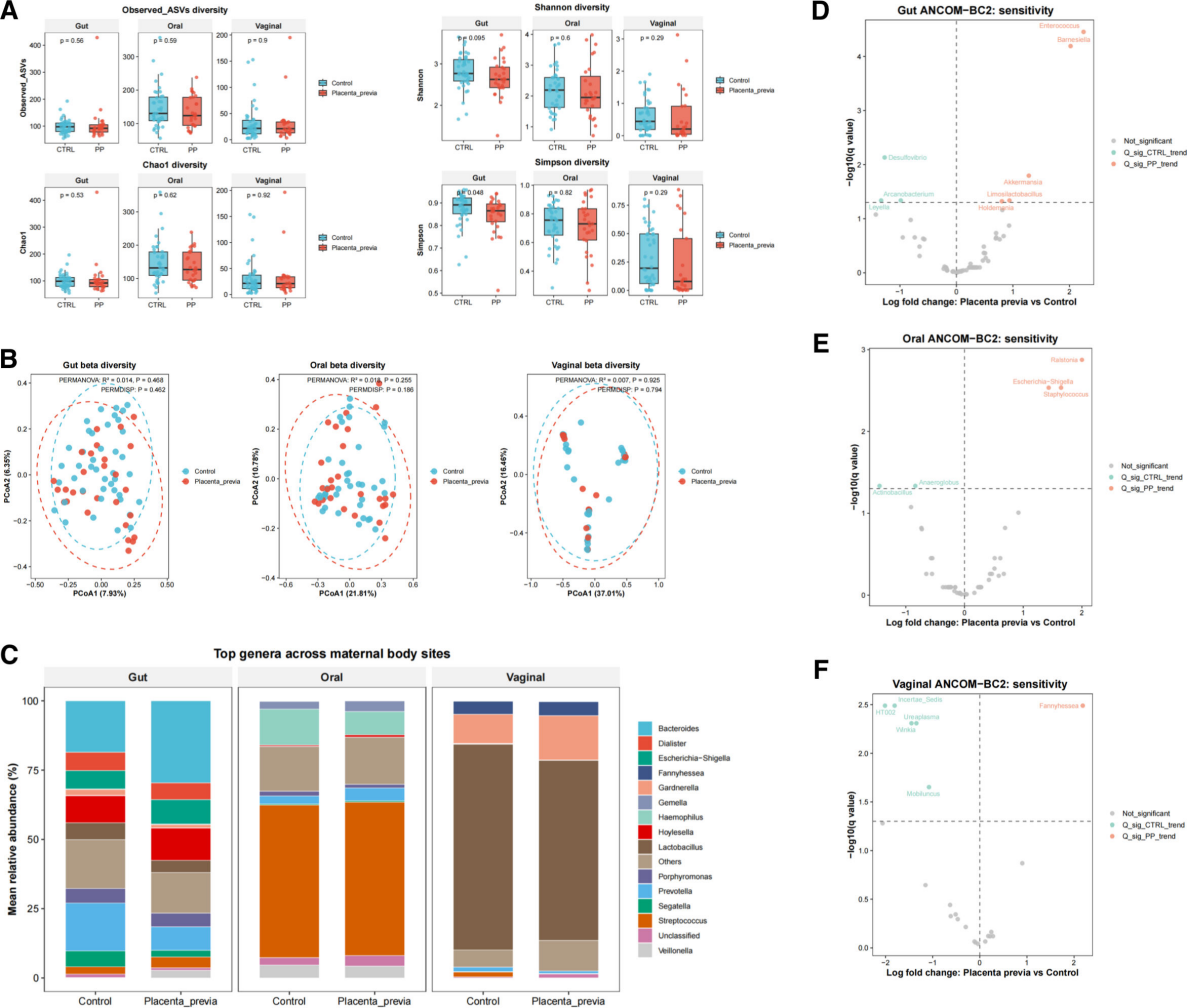
Maternal microbiota composition and diversity analyses in PP and CTRL pregnancies. (**A**) Alpha diversity analyses of gut, oral, and vaginal microbiota, including observed ASVs, Chao1 richness, Shannon diversity, and Simpson diversity indices. Overall microbial richness and diversity were largely comparable between groups across maternal body sites, although a modest reduction in gut Simpson diversity was observed in the PP group. (**B**) Beta diversity analyses based on weighted UniFrac distances visualized by PCoA. PERMANOVA analysis did not identify significant differences in overall microbial community composition between groups, and PERMDISP analysis showed no significant differences in within-group dispersion. (**C**) Relative abundance of dominant genera across gut, oral, and vaginal microbiota in PP and CTRL groups. The dominant microbial compositions of maternal body sites were generally preserved between groups. (**D through F**) Differential abundance analyses using ANCOM-BC2 with sensitivity filtering identified several site-specific microbial alterations associated with placenta previa. (**D**) Gut microbiota. (**E**) Oral microbiota. (**F**) Vaginal microbiota. Taxa enriched in the PP group and CTRL group are highlighted separately. Sensitivity analyses were performed using stricter prevalence-based filtering criteria to evaluate the robustness of differential abundance findings.

Alpha diversity analyses demonstrated largely comparable microbial richness and diversity between the two groups across all body sites (Fig. 2A). No significant differences were observed in observed ASVs, Chao1 richness, or Shannon diversity indices. A modest reduction in gut Simpson diversity was observed in the PP compared with CTRL subjects, suggesting subtle possible alterations in community evenness.

Beta diversity analysis based on weighted UniFrac distances further demonstrated substantial overlap between the two groups in gut, oral, and vaginal microbiota (Fig. 2B). PERMANOVA analysis did not identify significant differences in overall community composition, and PERMDISP analysis showed no significant differences in within-group dispersion.

Consistent with the diversity analyses, the dominant microbial compositions of the gut, oral cavity, and vagina were generally preserved between the PP and CTRL (Fig. 2C). In the gut microbiota, *Bacteroides*, *Prevotella*, and *Lactobacillus*-related taxa remained predominant in both groups. Oral microbiota were largely dominated by *Streptococcus* and *Haemophilus*, whereas *Lactobacillus* remained the major genus in vaginal samples across both groups.

Despite the absence of marked global differences in microbial diversity and dominant community composition, differential abundance analysis revealed several site-specific microbial alterations associated with placenta previa across gut, oral, and vaginal microbiota (Fig. 2D through F).

In gut microbiota, *Enterococcus*, *Barnesiella*, *Akkermansia*, and *Limosilactobacillus* demonstrated increased relative abundance in the PP, whereas *Desulfovibrio*, *Leyella*, and *Arcanobacterium* were relatively depleted. Several taxa retained similar directional trends following stricter prevalence-based sensitivity filtering, although statistical significance was attenuated.

Within oral microbiota, *Ralstonia*, *Escherichia-Shigella*, and *Staphylococcus* were enriched in the PP, whereas *Anaeroglobus* and *Actinobacillus* showed relative depletion compared with CTRL subjects. Similar directional trends were observed in sensitivity analyses with stricter prevalence filtering.

In vaginal microbiota, *Fannyhessea* demonstrated relative enrichment in PP subjects, whereas several low-prevalence taxa, including *Mobiluncus*, *Ureaplasma*, *Winkia*, *HT002*, and *Incertae_Sedis*-related taxa, showed depletion trends following prevalence-based sensitivity analysis.

Importantly, most identified taxa were low-abundance organisms and did not fulfill the strictest robustness criteria implemented in ANCOM-BC2. Therefore, these findings should be interpreted cautiously. Nevertheless, the partially consistent directional trends observed across sensitivity analyses suggest the possibility of subtle microbial compositional alterations associated with placenta previa rather than broad microbial ecosystem disruption.

### Microbial ecological networks were remodeled in placenta previa across maternal body sites

To further investigate whether placenta previa was associated with altered microbial ecological interactions despite relatively preserved global diversity, co-occurrence network analyses were performed for gut, oral, and vaginal microbiota in both groups.

**Fig 3:**
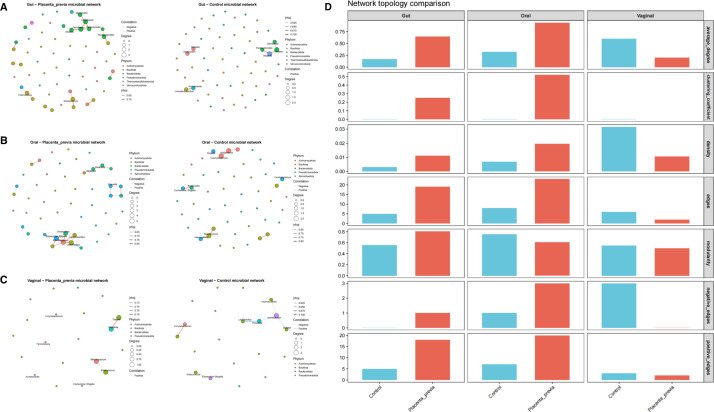
Microbial ecological network remodeling across maternal body sites in PP and CTRL pregnancies. (**A through C**) Co-occurrence microbial ecological networks of gut (**A**), oral (**B**), and vaginal (**C**) microbiota in PP and CTRL groups. Nodes represent microbial genera, and node size reflects network degree. Edge color indicates positive or negative correlations, and edge thickness corresponds to correlation strength. In the gut microbiota, the PP group exhibited a markedly more interconnected ecological network compared with CTRL subjects, with increased edge number, network density, average degree, and clustering coefficient. Hub taxa in the PP gut network included *Coprobacter*, *Odoribacter*, *Barnesiella*, and *Enterococcus*, whereas the CTRL network remained relatively sparse. Similarly, oral microbiota in the PP group demonstrated substantially increased network complexity compared with controls, characterized by higher connectivity and clustering. The oral PP network was organized around several highly connected taxa, including *Escherichia-Shigella*, *Lactobacillus*, *Bifidobacterium*, and *Bacteroides*. In contrast, vaginal microbiota in the PP group exhibited markedly reduced ecological connectivity, retaining only two positive associations (*Bifidobacterium–Enterococcus* and *Dialister–Prevotella*), whereas the CTRL vaginal network contained both positive and negative interactions, including a negative correlation between *Lactobacillus* and *Prevotella*. (**D**) Summary of microbial ecological network metrics across maternal body sites, including node number, edge number, network density, average degree, clustering coefficient, and modularity. Collectively, these analyses demonstrate substantial remodeling of microbial ecological architecture in placenta previa, with enhanced interaction complexity in gut and oral microbiota but simplified vaginal microbial networks.

In the gut microbiota, the PP exhibited a markedly more interconnected ecological network than the control group, characterized by increased edge number (19 vs 5), higher network density (0.011 vs 0.003), increased average degree (0.64 vs 0.17), and elevated clustering coefficient (0.25 vs 0) (Fig. 3A and D). Hub taxa in the placenta previa gut network included *Coprobacter*, *Odoribacter*, *Barnesiella*, and *Enterococcus*, whereas the control network was sparse and primarily centered around *Alistipes* and *Bilophila*. Most interactions in the placenta previa gut network were positive correlations, although one negative association between *Megasphaera* and *Negativicoccus* was observed.

Similarly, the oral microbiota in the PP demonstrated substantially increased network complexity compared with controls, including higher edge number (23 vs 8), density (0.020 vs 0.007), average degree (0.94 vs 0.33), and clustering coefficient (0.52 vs 0) (Fig. 3B and D). The oral network in placenta previa was organized around multiple highly connected taxa, including *Escherichia-Shigella*, *Lactobacillus*, *Bifidobacterium*, and *Bacteroides*. Positive associations dominated the oral microbial network, whereas a limited number of negative correlations involving *Tannerella*, *Haemophilus*, and *Lentimicrobium* were identified.

In contrast, the vaginal microbiota exhibited reduced ecological connectivity in the PP. Compared with controls, the placenta previa vaginal network showed decreased edge number (2 vs 6), lower density (0.011 vs 0.032), and reduced average degree (0.20 vs 0.60) (Fig. 3C and D). The control vaginal network contained both positive and negative interactions, including a negative correlation between *Lactobacillus* and *Prevotella*, whereas the placenta previa network retained only two positive associations (*Bifidobacterium–Enterococcus* and *Dialister–Prevotella*).

Collectively, these findings suggest that placenta previa is associated with substantial remodeling of microbial ecological architecture across maternal body sites. Notably, gut and oral microbial communities displayed enhanced interaction complexity, whereas vaginal microbial networks became markedly simplified.

### Placental SCFA alterations in placenta previa pregnancies

**Fig 4:**
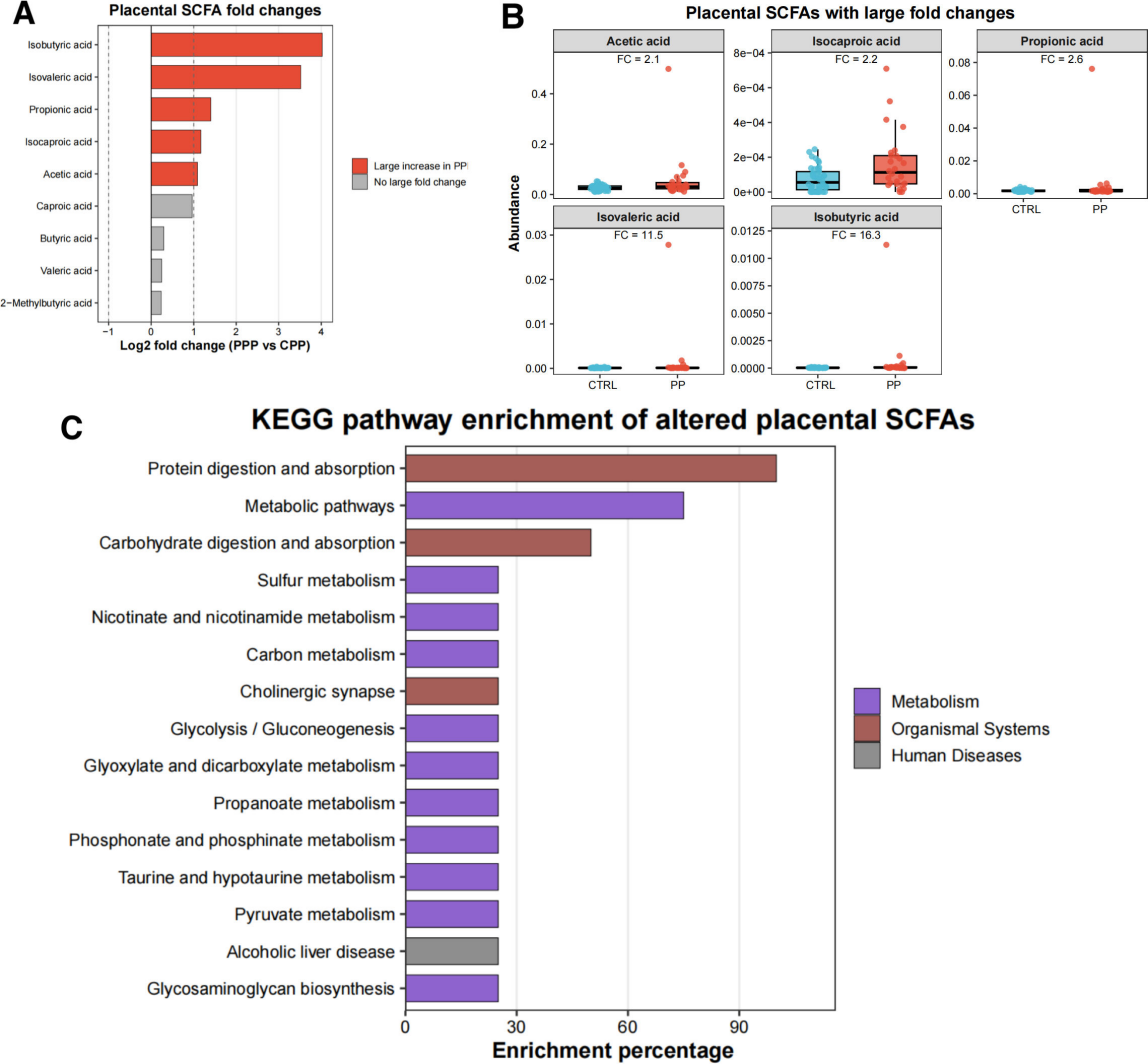
Placental SCFA alterations in PP and CTRL pregnancies. (**A**) Fold-change-based overview of placental SCFA alterations between PP and CTRL. Several SCFAs exhibited increased abundance trends in the PP, particularly isobutyric acid and isovaleric acid, followed by propionic acid, isocaproic acid, and acetic acid, whereas caproic acid, butyric acid, valeric acid, and 2-methylbutyric acid showed relatively limited alterations. (**B**) Individual comparison of placental SCFAs with a fold change >2 between groups. Acetic acid, isocaproic acid, propionic acid, isovaleric acid, and isobutyric acid demonstrated higher abundance trends in PP placentas, with isobutyric acid and isovaleric acid showing the most prominent increases. Data are presented as box plots with individual sample distributions. (**C**) KEGG pathway annotation analysis of altered placental SCFAs. Enriched pathways were mainly associated with nutrient absorption and metabolic regulation, including protein digestion and absorption, carbohydrate digestion and absorption, propanoate metabolism, glycolysis/gluconeogenesis, pyruvate metabolism, carbon metabolism, and cholinergic synapse-related pathways. These findings suggest coordinated metabolic remodeling and neuroactive signaling-associated alterations in PP placentas.

Placental targeted SCFA profiling revealed distinct metabolic alterations between the PP and CTRL. Fold-change-based analysis demonstrated that several SCFAs showed marked elevation in the PP, particularly isobutyric acid and isovaleric acid, which exhibited the largest increases, followed by propionic acid, isocaproic acid, and acetic acid (Fig. 4A). In contrast, caproic acid, butyric acid, valeric acid, and 2-methylbutyric acid showed relatively limited changes between groups.

To further visualize the metabolites with substantial alterations, metabolites with fold change >2 were selected for individual comparison (Fig. 4B). Among these, acetic acid, isocaproic acid, propionic acid, isovaleric acid, and isobutyric acid all demonstrated higher abundance trends in the PP. Notably, isobutyric acid and isovaleric acid exhibited the most prominent fold increases, suggesting pronounced remodeling of placental SCFA composition in PP placentas.

KEGG pathway annotation analysis further indicated that these altered placental SCFAs were mainly associated with pathways involved in nutrient absorption and metabolic regulation, including protein digestion and absorption, carbohydrate digestion and absorption, propanoate metabolism, glycolysis/gluconeogenesis, pyruvate metabolism, carbon metabolism, and cholinergic synapse-related pathways (Fig. 4C). Collectively, these findings suggest that placental SCFA alterations in PP may be linked to changes in metabolic homeostasis and neuroactive signaling-related processes.

### Placental transcriptomic remodeling

**Fig 5:**
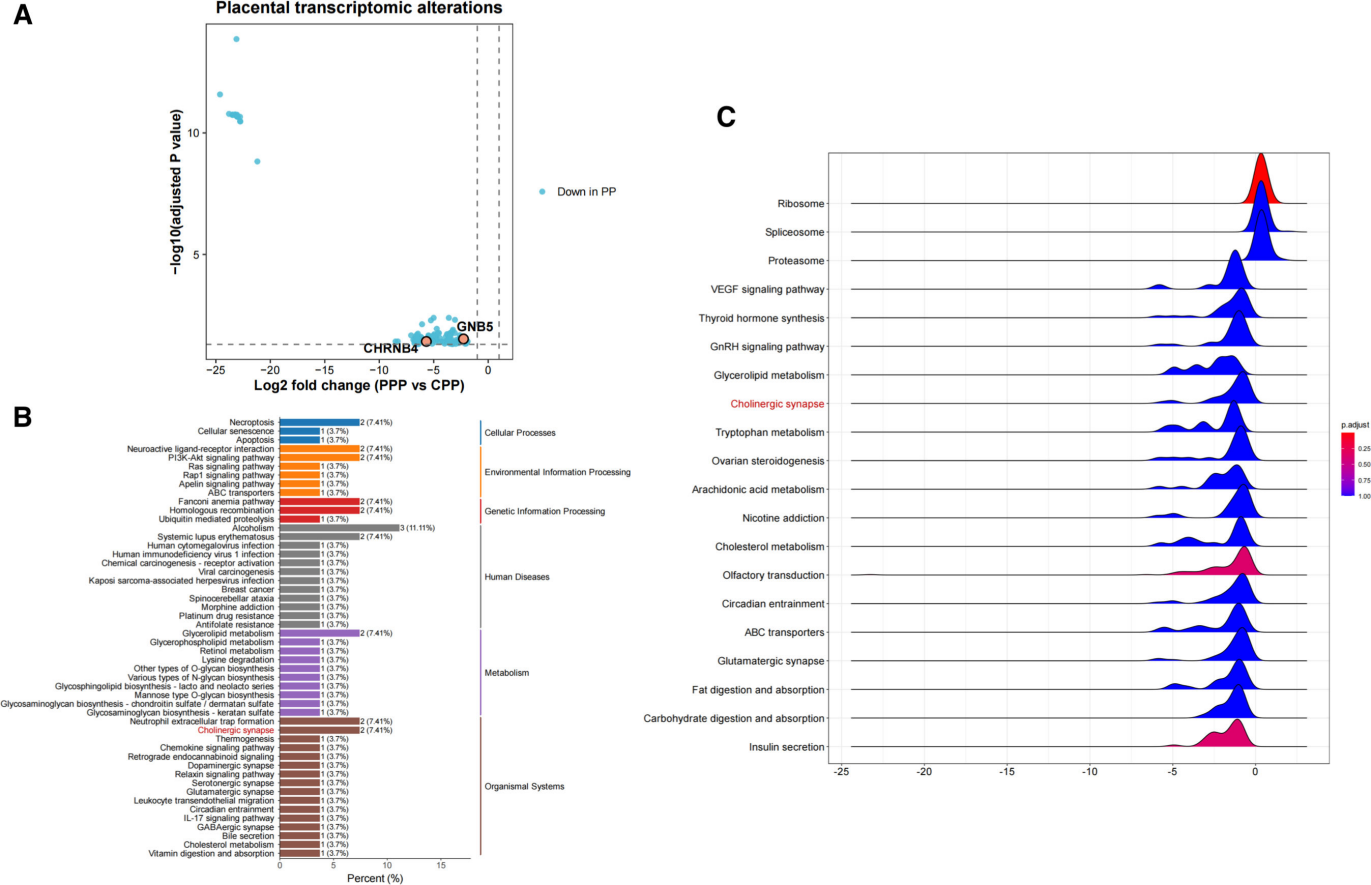
Placental transcriptomic alterations in PP and CTRL pregnancies. (**A**) Volcano plot of differentially expressed genes (DEGs) identified by placental RNA-seq analysis between PP and CTRL groups. Most significantly altered genes demonstrated downregulated expression patterns in PP placentas, indicating a predominance of suppressed transcriptional signals. (**B**) KEGG pathway enrichment analysis of differentially expressed genes. Enriched pathways included neuroactive ligand–receptor interaction, cholinergic synapse, glycerolipid metabolism, circadian entrainment, ABC transporters, PI3K-Akt signaling, and IL-17 signaling pathways, suggesting coordinated alterations in neuroactive, metabolic, and immune-related transcriptional programs. (**C**) Gene set enrichment analysis (GSEA) demonstrates pathway-level transcriptional alterations in PP placentas. Pathways associated with cholinergic synapse, glutamatergic synapse, circadian entrainment, cholesterol metabolism, carbohydrate digestion and absorption, and glycerolipid metabolism exhibited overall negative enrichment trends in PP placentas, supporting coordinated alterations in neuroactive and metabolic signaling pathways.

Placental transcriptomic profiling revealed transcriptomic alterations between the PP and CTRL. RNA-seq analysis demonstrated that most significantly altered genes were downregulated in the PP, suggesting a predominance of downregulated transcriptional signals (Fig. 5A).

KEGG enrichment analysis showed that the differentially expressed genes were mainly enriched in pathways related to neuroactive ligand–receptor interaction, cholinergic synapse, glycerolipid metabolism, circadian entrainment, and ABC transporters, as well as several immune- and signaling-associated pathways, including PI3K-Akt signaling and IL-17 signaling (Fig. 5B).

Consistent with the KEGG results, GSEA further demonstrated coordinated alterations in multiple pathway-level transcriptional programs. Pathways associated with cholinergic synapse, glutamatergic synapse, circadian entrainment, cholesterol metabolism, carbohydrate digestion and absorption, and glycerolipid metabolism showed overall negative enrichment trends in PP placentas (Fig. 5C), supporting coordinated alterations in neuroactive and metabolic signaling pathways.

### Associations among gut microbiota, placental SCFAs, and cholinergic signaling-related genes

**Fig 6:**
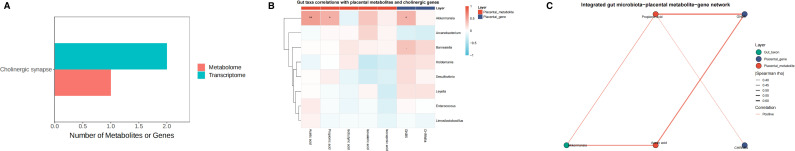
Integrated multi-omics analyses linking gut microbiota, placental SCFAs, and cholinergic signaling-related transcriptomic alterations in PP. (**A**) Comparative co-enrichment analysis of placental SCFA-associated metabolic pathways and placental transcriptomic pathways. Cholinergic synapse was identified as a shared enriched pathway across both omics layers, suggesting coordinated neuroactive signaling-related alterations in PP placentas. Representative cholinergic signaling-related genes, including GNB5 and CHRNB4, were subsequently selected for integrative analyses. (**B**) Correlation heatmap showing associations among altered gut microbial taxa, placental SCFAs, and cholinergic signaling-related genes. Positive and negative correlations were determined using Spearman correlation analysis. Notably, *Akkermansia* demonstrated positive correlations with acetic acid, propionic acid, and GNB5 expression. (**C**) Integrated association network illustrating potential relationships among gut microbial taxa, placental SCFAs, and cholinergic signaling-related genes in PP pregnancies. Nodes represent microbial taxa, metabolites, or genes, whereas edges indicate significant correlations. Collectively, these analyses suggest coordinated microbiota-associated metabolic and transcriptomic alterations linked to cholinergic synapse signaling in placenta previa.

To further investigate shared biological alterations across different omics layers, integrated co-enrichment analysis was subsequently performed. Comparative pathway enrichment demonstrated that the cholinergic synapse was enriched in both placental SCFA-associated metabolic pathways and placental transcriptomic pathways (Fig. 6A), suggesting that cholinergic signaling may represent a shared biological feature associated with PP.

Based on the shared cholinergic signaling pathway identified by integrated analyses, representative pathway-related genes were further explored. GNB5 and CHRNB4 were selected as cholinergic signaling-related genes for subsequent integrative analyses (Fig. 5A).

Given that SCFAs are primarily derived from gut microbial fermentation, subsequent integrative association analyses were focused mainly on gut microbial taxa. Correlation heatmap analysis demonstrated coordinated associations among gut microbial taxa, placental SCFAs, and cholinergic signaling-related genes (Fig. 6B). Notably, Akkermansia showed positive correlations with acetic acid, propionic acid, and GNB5 expression. Integrated network analysis further suggested potential associations linking altered gut microbes, placental SCFAs, and cholinergic signaling-related genes in PP (Fig. 6C).

## DISCUSSION

Recent interest in the maternal microbiota has revealed its potential involvement in various pregnancy-related complications (27–29). In the present multi-omics study, we comprehensively profiled maternal gut, oral, and vaginal microbiota together with placental SCFA and transcriptomic alterations in pregnancies complicated by placenta previa. To our knowledge, this represents the first integrated microbiome–metabolome–transcriptome analysis focused on placenta previa.

Rather than observing large-scale disruption of overall microbial diversity, we identified coordinated alterations in microbial ecological architecture, placental SCFA profiles, and placental transcriptomic programs. Notably, integrated pathway analyses demonstrated convergent enrichment of cholinergic synapse-related signaling across both placental SCFA-associated pathways and placental transcriptomic alterations. Furthermore, association analyses linked specific gut microbial taxa with placental SCFAs and cholinergic signaling-related genes. Collectively, these findings suggest the existence of a potential gut microbiota–placental metabolite–transcriptome axis associated with placenta previa, with gut microbiota-associated metabolic signaling emerging as the most prominent microbial feature identified in the present study.

Although alpha- and beta-diversity analyses demonstrated largely preserved global microbial composition across maternal body sites, microbial ecological network analyses revealed substantial remodeling of microbial interaction patterns in placenta previa. Among maternal microbial compartments, gut microbiota exhibited the most prominent ecological remodeling in placenta previa, characterized by markedly increased network connectivity and interaction complexity. Oral microbiota demonstrated similar alterations, whereas vaginal microbial networks became comparatively simplified.

These findings suggest that placenta previa may be associated not necessarily with broad microbial ecosystem disruption, but rather with altered microbial interaction architecture. Increasing evidence suggests that microbial ecological networks may provide additional biological information beyond taxonomic abundance alone, reflecting cooperative or competitive relationships that influence microbial community stability and host signaling (30, 31). Notably, several taxa with altered network connectivity in the present study, including *Akkermansia* (32), *Barnesiella* (33), and *Enterococcus* (34), have previously been associated with host metabolic and immune regulation.

Because obesity may influence gut microbial composition and host metabolism, maternal BMI was evaluated as a potential confounder. No significant difference in BMI was observed between groups. Importantly, most differentially abundant taxa identified in the present study were low-abundance organisms and did not satisfy the strictest robustness criteria across all sensitivity analyses. Therefore, these findings should be interpreted cautiously. Nevertheless, the partially consistent directional trends across analyses, together with consistent ecological network remodeling, suggest the presence of subtle but coordinated microbial ecological alterations associated with placenta previa.

Placental SCFA profiling further demonstrated coordinated metabolic alterations in placenta previa pregnancies. Several placental SCFAs, particularly isobutyric acid, isovaleric acid, propionic acid, isocaproic acid, and acetic acid, exhibited increased abundance trends in the placenta previa group. KEGG pathway annotation further indicated that these altered SCFAs were associated with pathways involved in carbohydrate metabolism, pyruvate metabolism, glycolysis/gluconeogenesis, nutrient absorption, and cholinergic synapse-related signaling.

SCFAs are increasingly recognized not only as microbial metabolic byproducts but also as signaling molecules capable of influencing host immune responses, epithelial integrity, energy metabolism, and neuroactive signaling pathways (35, 36). Previous studies have demonstrated that microbiota-derived metabolites can participate in maternal–fetal communication during pregnancy through the gut-placenta immune axis (13, 37). In the present study, the coordinated alteration of multiple placental SCFAs, together with their associations with specific gut microbial taxa, suggests the presence of altered microbiota-associated metabolic signaling in placenta previa.

Among the altered metabolites, acetic acid was of particular interest because it was linked to cholinergic synapse-related pathways in the integrated analyses. Although the precise biological significance of these alterations remains unclear, these findings raise the possibility that altered microbiota-associated SCFA metabolism may participate in placental signaling alterations associated with placenta previa.

One of the most notable findings of the present study was the consistent identification of cholinergic synapse-related signaling across multiple omics layers. Both placental SCFA-associated pathway analyses and placental transcriptomic analyses converged on cholinergic synapse signaling, suggesting that neuroactive signaling pathways may represent an important biological feature associated with placenta previa.

The cholinergic system has increasingly been recognized as an important regulator of immune homeostasis, inflammatory signaling, and tissue adaptation during pregnancy (38, 39). Previous studies have demonstrated that acetylcholine receptors are expressed in placental tissues and may participate in trophoblast proliferation, apoptosis, vascular regulation, and maternal–fetal immune communication (40–42).

In the present study, GNB5 and CHRNB4 were identified as representative cholinergic signaling-related genes associated with the shared enriched pathway. In addition, acetic acid and propionic acid were linked to cholinergic synapse-related metabolic pathways and demonstrated coordinated associations with gut microbial taxa and cholinergic signaling-related genes.

Although the biological relationships among gut microbial alterations, placental SCFAs, and cholinergic signaling remain incompletely understood, our integrated analyses raise the possibility that microbiota-associated metabolic signaling may be linked to placental neuroactive signaling alterations in placenta previa. Importantly, the current study does not establish causal relationships, and further mechanistic studies will be necessary to determine whether altered SCFA signaling contributes to placental cholinergic signaling alterations during pregnancy.

This study has several strengths. To our knowledge, this is the first study to comprehensively integrate maternal microbiota profiling across multiple body sites with placental SCFA metabolomics and placental transcriptomics in pregnancies complicated by placenta previa. By combining microbial ecological network analysis, placental metabolite profiling, transcriptomic pathway analysis, and multi-omics integration, the present study provided a systems-level characterization of microbiota-associated biological alterations in placenta previa. Notably, convergent enrichment of cholinergic synapse-related signaling across metabolomic and transcriptomic analyses strengthened the biological consistency of the multi-omics findings.

Several limitations should also be acknowledged. First, the present study was observational in nature, and causal relationships between maternal microbiota alterations and placenta previa cannot be established. In addition, microbiome, metabolomic, and transcriptomic profiling were performed at a single time point near delivery, precluding assessment of temporal dynamics throughout pregnancy. Second, although integrated analyses suggested potential associations among gut microbial taxa, placental SCFAs, and cholinergic signaling-related pathways, direct mechanistic validation was not performed. In addition, although the present study integrated multiple high-dimensional omics data sets, the relatively modest cohort size may limit statistical power and increase the possibility of unstable exploratory findings despite multiple testing correction procedures. Third, most differentially abundant microbial taxa identified in this study were low-abundance organisms and did not satisfy the strictest robustness criteria across all sensitivity analyses, warranting cautious interpretation. Fourth, microbiota profiling was performed using 16S rRNA gene sequencing, which lacks strain-level resolution and functional characterization compared with shotgun metagenomic sequencing. In addition, only a single placental tissue region from the maternal side was analyzed for each participant. Therefore, potential spatial heterogeneity of placental metabolomic and transcriptomic profiles may not have been fully captured. Finally, although the interval between deliveries was relatively limited, gestational age differed between groups, and residual confounding related to gestational timing cannot be completely excluded. Future studies incorporating mechanistic experiments, larger longitudinal cohorts, and higher-resolution multi-omics approaches will be important to further elucidate the role of gut microbiota-associated metabolic signaling in placenta previa.

### Conclusion

This integrated multi-omics study identified coordinated alterations in maternal microbiota ecology, placental short-chain fatty acid profiles, and placental transcriptomic programs in pregnancies complicated by placenta previa. Although global microbial diversity remained largely preserved, gut microbiota exhibited prominent ecological remodeling together with convergent metabolomic and transcriptomic enrichment of cholinergic synapse-related pathways. These findings support the existence of potential microbiota-associated metabolic and signaling alterations in placenta previa and provide a systems-level framework for future mechanistic investigation of microbiota–placenta interactions during pregnancy.

## Data Availability

The original data from this study are publicly accessible via the National Center for Biotechnology Information (NCBI) at https://www.ncbi.nlm.nih.gov/, under accession number PRJNA1470503.

## References

[1] Odendaal J, Black N, Bennett PR, Brosens J, Quenby S, MacIntyre DA. 2024. The endometrial microbiota and early pregnancy loss. Hum Reprod 39:638–646. doi:10.1093/humrep/dead274 38195891 PMC10988105

[2] Bayar E, Bennett PR, Chan D, Sykes L, MacIntyre DA. 2020. The pregnancy microbiome and preterm birth. Semin Immunopathol 42:487–499. doi:10.1007/s00281-020-00817-w 32797272 PMC7508933

[3] Benech N, Rolhion N, Sokol H. 2022. Gut microbiota reprogramming of tryptophan metabolism during pregnancy shapes host insulin resistance. Gastroenterology 162:1587–1589. doi:10.1053/j.gastro.2022.01.059 35247461

[4] Li Z, Zhang Y, Wang L, Deng TK, Chiu W-H, Ming W, Xu C, Xiao X. 2024. Microbiota of pregnancy, placenta and newborns in the third trimester: a randomized controlled study. Heliyon 10:e24698. doi:10.1016/j.heliyon.2024.e24698 38314279 PMC10837503

[5] Findeklee S, Costa SD. 2015. Placenta accreta and total placenta previa in the 19th week of pregnancy. Geburtshilfe Frauenheilkd 75:839–843. doi:10.1055/s-0035-1557763 26366004 PMC4554505

[6] Zhou B, Lian J, Wang Y, Yang Y, Bai H, Wu S. 2025. Development and validation of a model to preoperatively predict the risk of placenta accreta spectrum in women with placenta previa. Adv Clin Exp Med 34:1145–1153. doi:10.17219/acem/191828 39675014

[7] Hu H, Wang L, Gao J, Chen Z, Chen X, Tang P, Zhong Y. 2024. Risk factors of severe postpartum hemorrhage in pregnant women with placenta previa or low-lying placenta: a retrospective cohort study. BMC Pregnancy Childbirth 24:674. doi:10.1186/s12884-024-06876-3 39407169 PMC11475889

[8] White A, Malik M, Pruszynski JE, Do QN, Spong CY, Herrera CL. 2025. Contemporary placenta accreta spectrum disorder incidence and risk factors. Obstet Gynecol 145:665–673. doi:10.1097/AOG.0000000000005919 40245410 PMC12278684

[9] Landsverk E, Westvik-Johari K, Wennerholm U-B, Bergh C, Kyhl F, Spangmose AL, Vassard D, Pinborg A, Rönö K, Gissler M, Petersen SH, Opdahl S. 2025. Risk of placenta previa in assisted reproductive technology: a Nordic population study with sibling analyses. PLoS Med 22:e1004536. doi:10.1371/journal.pmed.1004536 39899615 PMC11835333

[10] Campisciano G, Zanotta N, Quadrifoglio M, Careri A, Torresani A, Cason C, De Seta F, Ricci G, Comar M, Stampalija T. 2023. The bacterial DNA profiling of chorionic villi and amniotic fluids reveals overlaps with maternal oral, vaginal, and gut microbiomes. Int J Mol Sci 24:2873. doi:10.3390/ijms24032873 36769194 PMC9917689

[11] Huang L, Cai M, Li L, Zhang X, Xu Y, Xiao J, Huang Q, Luo G, Zeng Z, Jin C, Jin Y, He J, Yang W. 2021. Gut microbiota changes in preeclampsia, abnormal placental growth and healthy pregnant women. BMC Microbiol 21:265. doi:10.1186/s12866-021-02327-7 34607559 PMC8489045

[12] Han YW, Redline RW, Li M, Yin L, Hill GB, McCormick TS. 2004. Fusobacterium nucleatum induces premature and term stillbirths in pregnant mice: implication of oral bacteria in preterm birth. Infect Immun 72:2272–2279. doi:10.1128/IAI.72.4.2272-2279.2004 15039352 PMC375172

[13] Giugliano S, Gatti A, Rusin M, Schorn T, Pimazzoni S, Calanni-Pileri M, Fraccascia V, Carloni S, Rescigno M. 2025. Maternal gut microbiota influences immune activation at the maternal-fetal interface affecting pregnancy outcome. Nat Commun 16:4326. doi:10.1038/s41467-025-58533-8 40346042 PMC12064790

[14] Yang P, Li Z, Tye KD, Chen Y, Lu T, He Z, Zhou J, Xiao X. 2020. Effects of an orally supplemented probiotic on the autophagy protein LC3 and Beclin1 in placentas undergoing spontaneous delivery during normal pregnancy. BMC Pregnancy Childbirth 20:216. doi:10.1186/s12884-020-02905-z 32295534 PMC7161261

[15] Junaid M, Ahmad A, Hu Z, Xu M, Shi X, Qu N, Du T, Ding H, Zhu Y. 2026. The maternal and infant gut microbiome: implications for pregnancy outcomes, immune development, and health in the first 1000 days. J Transl Med 24:449. doi:10.1186/s12967-026-07846-3 41736017 PMC13040708

[16] Huang J, Zhang Y, Zheng W, Li G. 2026. Gut microbiota contributes to gestational diabetes mellitus by interfering with bile acid metabolism and resistin. Front Cell Infect Microbiol 16:1675560. doi:10.3389/fcimb.2026.1675560 41778019 PMC12950734

[17] Du H, Lin Q, He X, Yang B, Huang Y, Li Q, Wang Y, Wen R, Lin W, Li S, Zheng L, Ou Z. 2026. Dynamic involvement of the core gut microbiome XNP_Guild1 in the evolution of gestational diabetes mellitus. Gut Microbes 18:2623353. doi:10.1080/19490976.2026.2623353 41620634 PMC12867416

[18] Choo JM, Leong LEX, Rogers GB. 2015. Sample storage conditions significantly influence faecal microbiome profiles. Sci Rep 5:16350. doi:10.1038/srep16350 26572876 PMC4648095

[19] Gorzelak MA, Gill SK, Tasnim N, Ahmadi-Vand Z, Jay M, Gibson DL. 2015. Methods for improving human gut microbiome data by reducing variability through sample processing and storage of stool. PLoS One 10:e0134802. doi:10.1371/journal.pone.0134802 26252519 PMC4529225

[20] Griffiths RI, Thomson BC, James P, Bell T, Bailey M, Whiteley AS. 2011. The bacterial biogeography of British soils. Environ Microbiol 13:1642–1654. doi:10.1111/j.1462-2920.2011.02480.x 21507180

[21] Bolyen E, Rideout JR, Dillon MR, Bokulich NA, Abnet CC, Al-Ghalith GA, Alexander H, Alm EJ, Arumugam M, Asnicar F, et al.. 2019. Reproducible, interactive, scalable and extensible microbiome data science using QIIME 2. Nat Biotechnol 37:852–857. doi:10.1038/s41587-019-0209-9 31341288 PMC7015180

[22] Amir A, McDonald D, Navas-Molina JA, Kopylova E, Morton JT, Zech Xu Z, Kightley EP, Thompson LR, Hyde ER, Gonzalez A, Knight R. 2017. Deblur rapidly resolves single-nucleotide community sequence patterns. mSystems 2:e00191-16. doi:10.1128/mSystems.00191-16 28289731 PMC5340863

[23] Bokulich NA, Kaehler BD, Rideout JR, Dillon M, Bolyen E, Knight R, Huttley GA, Gregory Caporaso J. 2018. Optimizing taxonomic classification of marker-gene amplicon sequences with QIIME 2’s q2-feature-classifier plugin. Microbiome 6:90. doi:10.1186/s40168-018-0470-z 29773078 PMC5956843

[24] Love MI, Huber W, Anders S. 2014. Moderated estimation of fold change and dispersion for RNA-seq data with DESeq2. Genome Biol 15:550. doi:10.1186/s13059-014-0550-8 25516281 PMC4302049

[25] Anderson MJ. 2006. Distance-based tests for homogeneity of multivariate dispersions. Biometrics 62:245–253. doi:10.1111/j.1541-0420.2005.00440.x 16542252

[26] Lin H, Peddada SD. 2024. Multigroup analysis of compositions of microbiomes with covariate adjustments and repeated measures. Nat Methods 21:83–91. doi:10.1038/s41592-023-02092-7 38158428 PMC10776411

[27] Flores Ventura E, Lane JA, Turjeman S, Vidra N, Weiss GA, Gross G, Chang CY, Koren O. 2025. ILSI Europe perspective review: site-specific microbiota changes during pregnancy associated with biological consequences and clinical outcomes: opportunities for probiotic interventions. Gut Microbes 17:2501186. doi:10.1080/19490976.2025.2501186 40397816 PMC12101587

[28] Xu S, Xiong J, Qin X, Ma M, Peng Y, Cheng J, Nie X, Fan X, Deng Y, Ju Y, Liu J, Zhang L, Liu B, Zhang Y, Li L. 2025. Association between gut microbiota and perinatal depression and anxiety among a pregnancy cohort in Hunan, China. Brain Behav Immun 125:168–177. doi:10.1016/j.bbi.2024.12.150 39736365

[29] Li X, Xie H, Chao JJ, Jia YH, Zuo J, An YP, Bao YR, Jiang X, Ying H. 2023. Profiles and integration of the gut microbiome and fecal metabolites in severe intrahepatic cholestasis of pregnancy. BMC Microbiol 23:282. doi:10.1186/s12866-023-02983-x 37784030 PMC10546765

[30] Weng L, Cui Y, Jian W, Zhang Y, Pang L, Cao Y, Zhou Y, Liu W, Lin H, Tao Y. 2025. Inter-kingdom interactions and environmental influences on the oral microbiome in severe early childhood caries. Microbiol Spectr 13:e02518-24. doi:10.1128/spectrum.02518-24 40243315 PMC12131756

[31] Pan Z, Chen Y, Zhou M, McAllister TA, Mcneilly TN, Guan LL. 2024. Linking active rectal mucosa-attached microbiota to host immunity reveals its role in host-pathogenic STEC O157 interactions. ISME J 18:wrae127. doi:10.1093/ismejo/wrae127 38984791 PMC11304501

[32] Zhao Y, Yang H, Wu P, Yang S, Xue W, Xu B, Zhang S, Tang B, Xu D. 2024. *Akkermansia muciniphila*: a promising probiotic against inflammation and metabolic disorders. Virulence 15:2375555. doi:10.1080/21505594.2024.2375555 39192579 PMC11364076

[33] Gou Y, Lin F, Dan L, Zhang D. 2024. Exposure to toluene diisocyanate induces dysbiosis of gut-lung homeostasis: involvement of gut microbiota. Environ Pollut 363:125119. doi:10.1016/j.envpol.2024.125119 39414067

[34] Duan Y, Chu H, Brandl K, Jiang L, Zeng S, Meshgin N, Papachristoforou E, Argemi J, Mendes BG, Wang Y, Su H, Sun W, Llorente C, Hendrikx T, Liu X, Hosseini M, Kisseleva T, Brenner DA, Bataller R, Ramachandran P, Karin M, Fu W, Schnabl B. 2021. CRIg on liver macrophages clears pathobionts and protects against alcoholic liver disease. Nat Commun 12:7172. doi:10.1038/s41467-021-27385-3 34887405 PMC8660815

[35] Zurdo-López M, Sagredo Del Rio M, Cháfer Rudilla M, Ibarra A, Doncel-Pérez E. 2026. Microbiota and Guillain-Barré syndrome: role of microbial metabolites, biomarkers, and emerging therapeutic strategies. Front Neurol 17:1815899. doi:10.3389/fneur.2026.1815899 42111070 PMC13149125

[36] Hays KE, Pfaffinger JM, Ryznar R. 2024. The interplay between gut microbiota, short-chain fatty acids, and implications for host health and disease. Gut Microbes 16:2393270. doi:10.1080/19490976.2024.2393270 39284033 PMC11407412

[37] Brown JA, Amir M, Yu S, Wong DSH, Gu J, Balaji U, Parkhurst CN, Hong S, Hart LR, Carrow HC, Bah MA, Ananthanarayanan A, Sanidad KZ, Lyu M, Siddikova A, Santos MLS, Serganova I, Diehl GE, Anrather J, Inohara N, Sonnenberg GF, Pascual V, Zeng MY. 2026. Gut microbiota promotes immune tolerance at the maternal-fetal interface. Cell 189:196–214. doi:10.1016/j.cell.2025.11.022 41412123 PMC12904261

[38] Pavía J, Muñoz M, Jiménez E, Martos F, Gonzalez-Correa JA, De la Cruz JP, Garcia V, Sanchez de la Cuesta F. 1997. Pharmacological characterization and distribution of muscarinic receptors in human placental syncytiotrophoblast brush-border and basal plasma membranes. Eur J Pharmacol 320:209–214. doi:10.1016/s0014-2999(96)00889-8 9059856

[39] Bhuiyan MB, Murad F, Fant ME. 2006. The placental cholinergic system: localization to the cytotrophoblast and modulation of nitric oxide. Cell Commun Signal 4:4. doi:10.1186/1478-811X-4-4 16686954 PMC1481520

[40] Lips KS, Brüggmann D, Pfeil U, Vollerthun R, Grando SA, Kummer W. 2005. Nicotinic acetylcholine receptors in rat and human placenta. Placenta 26:735–746. doi:10.1016/j.placenta.2004.10.009 16226123

[41] Sastry BV. 1997. Human placental cholinergic system. Biochem Pharmacol 53:1577–1586. doi:10.1016/s0006-2952(97)00017-8 9264309

[42] Wedn AM, El-Bassossy HM, Eid AH, El-Mas MM. 2021. Modulation of preeclampsia by the cholinergic anti-inflammatory pathway: Therapeutic perspectives. Biochem Pharmacol 192:114703. doi:10.1016/j.bcp.2021.114703 34324867

